# Provision of Palliative Care during the COVID-19 Pandemic: A Systematic Review of Ambulatory Care Organizations in the United States

**DOI:** 10.3390/medicina57101123

**Published:** 2021-10-18

**Authors:** Cristian Lieneck, Jose Betancourt, Cynthia Daemen, Rhiannon Eich, Elisabeth Monty, Mindy Jo Petty

**Affiliations:** 1 School of Health Administration, Texas State University, San Marcos, TX 78666, USA; jose.a.betancourt@txstate.edu; 2 St. David’s School of Nursing, Texas State University, Round Rock, TX 78665, USA; cyndaemen@txstate.edu (C.D.); rme67@txstate.edu (R.E.); esm104@txstate.edu (E.M.); mjp207@txstate.edu (M.J.P.)

**Keywords:** palliative care, hospice care, ambulatory care, outpatient care, COVID-19, pandemic

## Abstract

*Background and objectives:* Ambulatory (outpatient) healthcare organizations continue to respond to the COVID-19 global pandemic using an array of initiatives to sustain a continuity of palliative care. Continuance of palliative care during major crises has been previously accomplished; however, the global pandemic presents new challenges to the US healthcare industry. *Materials and methods:* This systematic review queried four research databases to identify applicable studies related to the provision of palliative care during the pandemic in outpatient organizations within the United States. *Results:* There are two primary facilitators for the ongoing provision of palliative care for the outpatient segment of the United States healthcare industry: technology and advanced care planning. Researchers also identified two primary barriers in the outpatient setting impacting the continuance of palliative care: lack of resources and accessibility to care. *Conclusions:* This systematic review identified facilitators and barriers for palliative care initiatives in the United States that can further assist future outpatient (ambulatory care) providers at a global level as the pandemic and associated public health initiatives continue.

## 1. Introduction

### 1.1. Rationale

The sustainability of palliative healthcare to patients in need in the United States has been profoundly impacted by the current COVID-19 global pandemic [1]. Quality of care concerns continue to exist despite the pervasive spread of COVID-19 throughout the US. Although imperfect, unique responses were developed within telemedicine platforms to mitigate disruptions broadly and effectively in care and treatment modalities [2]. Similar to the healthcare industry’s demonstrated ability to rapidly acclimate and change outpatient physical distancing care protocols [3], similar initiatives are required for palliative care in the outpatient setting.

The American Medical Association (AMA) defines palliative care as “that which relieves suffering and improves quality of life for people with serious illnesses, no matter whether they can be cured” [4]. Regardless of diagnosis, this industry service and related level of care is an important initiative to be continued throughout the COVID-19 pandemic. Existent in both in-and outpatient organizational settings, specific challenges, and related public health initiatives exist for outpatient (ambulatory) care organizations and the sustainability of palliative care.

The advent of the pandemic presented significant challenges to medical providers and their healthcare organizations as they sought innovative manners to continue administering to their patient population [5]. Conversely, the pandemic provided outpatient clinicians with certain opportunities to exhibit innovative solutions to these challenging hurdles [6]. History will likely label the COVID-19 pandemic a “disrupter” which served as a catalyst that accelerated the utility of such certain technologies such as telemedicine in the healthcare arena. Providers in the palliative care domain were forced to find innovative techniques to connect with their patients such as live video conferencing sessions and audio-only teleconferences involving multiple patient providers. Thus far, there exists limited research focused on how the current pandemic has both benefited and impeded the continuance of palliative care in the United States. This review differs from prior studies that explore the impact of COVID-19 on the delivery of healthcare because it focuses specifically on patients requiring palliative care in the outpatient (ambulatory care) healthcare organizations located within the United States. While unique, findings from the US healthcare system may benefit other industries to sustain quality palliative care provisions during the pandemic.

The paper is structured as follows: Section 2 describes the review’s materials and methods, including eligibility criteria and the search process; Section 3 describes the results (article exclusion, bias, and identification of constructs); Section 4 provides a discussion of the results obtained in the review, leading to the conclusions.

### 1.2. Objectives

The objective of this systematic review was to determine underlying constructs regarding facilitators and barriers experienced by ambulatory care organizations throughout the COVID-19 pandemic during the provision of palliative care. Although the healthcare industry in general was significantly impacted on various levels, our study team strived to answer the following question: “What impact has the current COVID-19 pandemic had on the provision of palliative care to those suffering from either an acute or a chronic condition and in need of these services?” Throughout the pandemic, telemedicine utilization increased on an unprecedented scale as healthcare providers across the United States sought to stem the deleterious effects of COVID-19. Was the same observed in the provision of palliative care? What were some of the additional “lessons learned” which could be captured by the entire palliative care domain as “best practices” which could be considered for implementation during the next pandemic (facilitators to palliative care sustainability)? These are but a few questions that our study team sought to answer. Only through this methodology can current best practices be captured for review and evaluated for successful outcomes. Conversely, practices that were incorporated as a result of the pandemic, but which had poor outcomes, can also serve as valuable tools for the palliative care industry to learn from (barriers to palliative care sustainability). Regardless of the outcome, the focus of our study was to examine each of these outcomes through an extensive review of the literature, categorize both facilitators and barriers to the provision of palliative care in ambulatory settings in the United States, and, on the basis of our findings, inform the community for follow-on sustainability practices for care.

### 1.3. Introduction Summary

The research findings demonstrate two underlying constructs (themes) supporting the facilitation of palliative care: technology and advanced care planning. This finding suggests methods in which additional outpatient clinics and related organizations may further enhance their provision of palliative care efforts during the pandemic. Furthermore, additional, barrier constructs (themes) were also identified as challenges to the continuity of palliative care: lack of resources and accessibility to care. Here, ambulatory care organizations can work to improve upon such barriers help enhance quality of care.

## 2. Materials and Methods

### 2.1. Eligibility Criteria

Studies for this review were focused specifically on the delivery or palliative care in ambulatory (outpatient) care organizations during the COVID-19 global pandemic. Publications entered into the review process were published in quality peer-reviewed and/or academic journals. The research team immediately noticed that many articles focused on the adaptive processes involved in the provision of palliative care during the pandemic yet did not include specific patient outcomes [7,8]. As a result, patient outcome was not a requirement for inclusion in the review process. An assessment of strength of evidence was conducted for each article using the Johns Hopkins evidence-based practice rating scale (JHNEBP), and only articles focused on outpatient organizations in the United States with a publication date between 1 January 2020 through 1 March 2021 were included in the review.

### 2.2. Information Sources

Four research databases were queried by the researchers to identify articles focusing on facilitators and barriers for the provision of palliative care during the pandemic:

- Academic Search Complete,
- MEDLINE Complete,
- Complementary Index,
- Health Source: Nursing/Academic Edition.

The database search was conducted from 15–20 April 2021.

### 2.3. Search

The research team utilized an aggressive search approach in order to identify articles that specifically focused on nonhospital, ambulatory care organizations in an attempt to identify underlying constructs related to delivery of palliative care during the pandemic. In addition to the article publication date range requirement, the use of Medial Subject Headings (MeSH) was utilized in order to identify all viable terminology to be incorporated in the search string. Both the ambulatory care and the palliative care review variables were investigated in the National Library of Medicine’s controlled vocabulary thesaurus, which indexes research articles for PubMed (MEDLINE). Once all related terms were identified for ambulatory care and palliative care (i.e., all equivalent “exploding terms” in the MeSH thesaurus) the research team conducted multiple iterations of searches using various Boolean operators for all review variables in an attempt to identify the highest review sample.

The final search string utilized in the review was as follows: (“ambulatory care” OR “outpatient care” OR “outpatient services” OR “urgent care” OR “clinic visits”) AND (“palliative care” OR “palliative supportive care” OR “palliative care medicine” OR “hospice and palliative care nursing” OR “palliative care nursing”) AND (“COVID-19” OR “coronavirus” OR “2019-nCoV” OR “SARS-CoV-2” OR “CoV-19”). Articles identified by the search string were included in the review process if they met the aggressive article publication date range, were available in the English language, and were published in academic/peer-reviewed journals.

### 2.4. Initial Study Selection

The review process was guided by the Preferred Reporting Items for Systematic Reviews and Meta-Analysis (PRISMA). Five of the six researchers participated in the initial database search and identification of articles to be included in the study. While many of the citations identified in the researchers’ library database were not initially available in full text, multiple researchers were able to utilize alternate library research databases to identify all necessary manuscripts identified by the initial search string by utilizing their adjunct faculty credentials with respective neighboring academic institutions where they also teach. By not including “full text only” in the search criteria, this action enabled a maximum number of potential articles to be available in the review process. A reference manager software program was utilized to maintain all citations/manuscripts identified by the research team’s search.

While the research team members were all associated with the same academic institution, the team’s interprofessional relationships involved significant geographic distancing between academic units. Therefore, multiple team meetings were conducted virtually using MS Teams and Zoom webinars throughout the entire review process. All six team members participated in the article review process (full text), to include initial article screening and underlying construct coding. This process was conducted by utilizing a team reading assignment (Table 1) and a cloud-based MS Excel literature review matrix via MS Teams. Each article was reviewed by three research team members, with two team members reviewing all articles in the sample.

Upon completion of the full-text review process, additional (virtual) team meetings were conducted to ensure a consensus regarding the underlying construct coding of articles demonstrating facilitators and barriers for the provision of palliative care experienced during the pandemic. There were only two initial disagreements among the team members and this process, and resolution regarding the literature review matrix and underlying themes identified was resolved among the group with 100% agreement in these two instances.

## 3. Results

### 3.1. Study Selection/Exclusion

**Figure 1:**
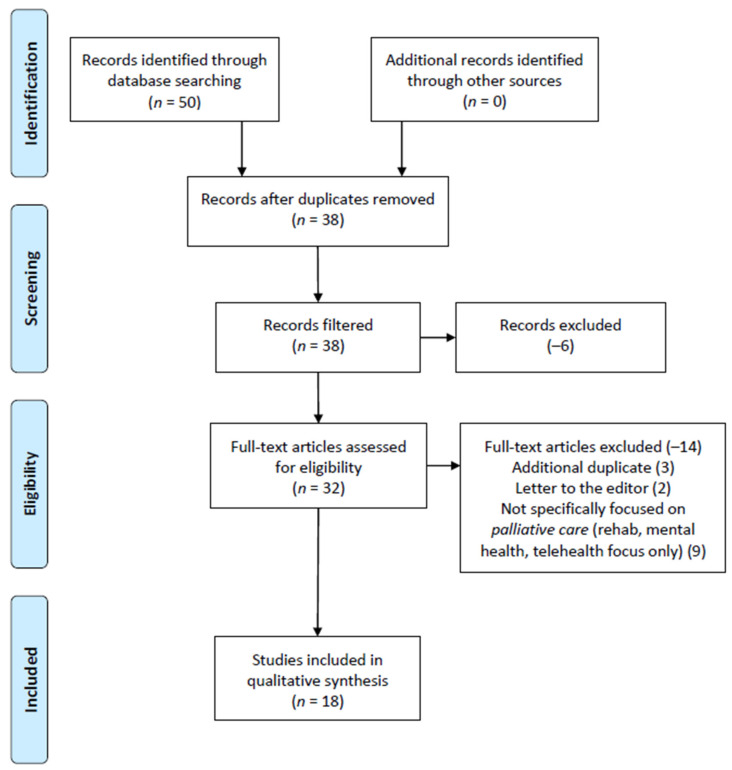
Preferred Reporting Items for Systematic Reviews and Meta-Analysis (PRISMA) figure that demonstrates the study selection process.

Figure 1 demonstrates the study selection and follow-on exclusion process, initially identifying 50 articles from all four research databases. Twelve duplicates were identified and removed across the entire search, and the study’s filtering process removed six articles from the initial research database query.

In addition to removing six articles for not meeting the study criteria, the full-text review of the remaining articles resulted in an additional 12 articles being excluded from the review. These articles were removed for the following reasons:

(a) Additional duplicate article identified (three articles),
(b) Letter to the editor (2 articles),
(c) Article not focused on palliative care provision, but rather facilitation initiatives related to rehabilitation services, mental health care, and telehealth resources (7 articles).

Upon completion of the review, a total of 18 articles were included in the review.

### 3.2. Study Characteristics

Reviews entailed a systematic approach in identifying underlying characteristics associated with the provision of palliative care initiatives utilized to date during the global pandemic, specific to outpatient/ambulatory care organizations. In addition to the JHNEBP study design analysis, both facilitators and barriers with regard to palliative care initiatives during the pandemic are summarized in Table 2.

### 3.3. Risk of Bias

The JHNEBP quality indicator frequencies from the sample are shown in Table 3. While it is preferred that research articles with strength of evidence ratings of level 1 and/or 2 are utilized in any systematic review, the researchers immediately identified a lack of published research in this segment healthcare industry to date. The majority of articles in the sample were classified as JHNEBP level 4 (67%) due to their findings and evidence that focused primarily upon the article authors’ provision of palliative care according to their own outpatient organizational experiences during the pandemic.

### 3.4. Additional Analysis

**Figure 2:**
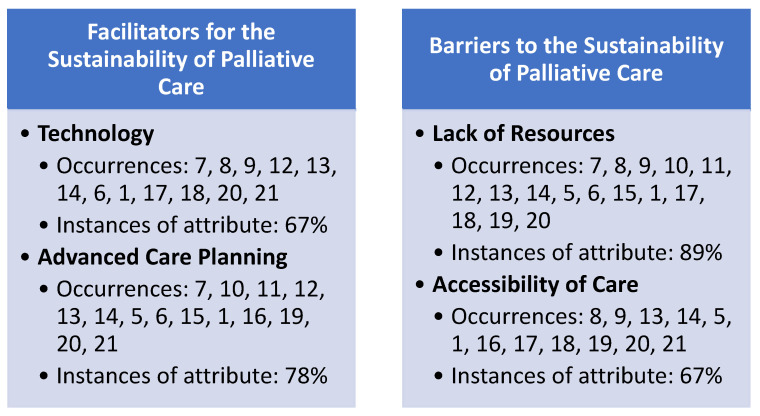
Identified themes (constructs) identified as facilitators and barriers for the sustainability of palliative care during the COVID-19 pandemic.

Results of the research team’s consensus meetings demonstrated three facilitator and three barrier themes identified in the literature to support the provision of palliative care in the ambulatory care segment of the industry during the pandemic (Figure 2). Findings are not mutually exclusive to only a facilitator or barrier theme, as several articles demonstrated both constructs upon review.

## 4. Discussion

### 4.1. Summary of Evidence

The sustainability of palliative care to the outpatient population in the United States has undergone a transformational change throughout the COVID-19 pandemic. The pandemic served as a catalyst for things such as the increased use of telemedicine between patient and provider, increased collaboration among providers, and increased facilitation of home healthcare, particularly those seeking to avoid hospitals for fear of exposure to the virus [2,6,14]. The literature has illustrated how human ingenuity and the need to find solutions, given the barriers to the provision of palliative care, produced unique solutions which should be institutionalized as common practice. A number of facilitators and barriers have been identified and focused on for further improvement and implementation to refine existing palliative care delivery approaches.

The research team identified four primary themes (constructs) associated with both facilitators and barriers influencing the provision of palliative care in the United States during the pandemic. Facilitator variables identified were technology (67% occurrence) and advanced care planning (78% occurrence). Likewise, identified barriers to the provision of palliative care and percentage of attribute occurrence include lack of resources (89% occurrence) and accessibility of care (67% occurrence). Within-construct sub-variables regarding facilitators and barriers for provision of palliative care were also able to be identified by the study team, indicated by the article (reference) numbers in Figure 2.

### 4.2. Palliative Care Facilitator: Technology

The shutdown of specialized healthcare services due to COVID-19 left vulnerable populations such as palliative care patients struggling to find necessary therapies and overall sustainable access to care. Telehealth, which were previously relied on infrequently, became the primary platform for healthcare delivery. The cessation of routine care at inpatient facilities (to include palliative care) meant that primary care physicians were taking on the responsibility for assessments and treatments in this population. Providing palliative care education and training for these providers and their subsequent use of telehealth resulted in a breakthrough for the delivery of patient care [9,12,17,21]. Such a breakthrough also included provider liability and best practices related to therapeutic medication diversion in the outpatient setting [17,21].

In addition to telehealth medicine for primary care visits, the literature suggested that, to mitigate the spread of COVID-19, pandemic plans were necessary for the continued health and safety of our population. Disaster plans can help keep high-risk patients safe, reduce the spread of the virus, and still be able to offer necessary healthcare via the use of technology. These plans included palliative care and grief resources, as well as implementing a triage system to get those high-risk patients seen first [1,6,13,14,20].

The use of telehealth and related communication methods with patients can present challenges to the provision of health as identified by the research team, beyond this study. Important to note, disparities surrounding healthcare technologies (outside of just palliative care) do exist [22]. Specific challenges may include access to broadband internet in rural areas [22,23], socioeconomic factors [24], and even health literacy and education challenges [25]. These challenges and others are to be continuously addressed to help enable technology, an identified review construct in this study, to support the continuity of palliative care.

### 4.3. Palliative Care Facilitator: Advanced Care Planning

Advanced planning facilitated patients in need of palliative care services to be prioritized within the challenges presented by COVID-19. In the more traditional interpretation of advanced care planning, patients who were working closely with their physician and care team may have already identified palliative care as the next progression in their care plan [12,20]. These patients were able to be connected to variable resources that were still available despite many of the barriers in the outpatient setting [7,11,12,13,15,16,19,20].

To transition to a disaster/pandemic plan, several organizations created and relied upon an assortment of triage tools to determine in advance how patients would best be served [1,5,6,7,14,15,20]. While palliative care teams were unable provide services to every patient that qualified, advanced planning removed many of the barriers and sought to implement creative solutions to care for the greatest number possible, while still maintaining safety for providers and being cognizant of resources.

### 4.4. Palliative Care Barrier: Lack of Resources

A lack of healthcare resources was a major barrier to the sustainability of palliative care services during the pandemic. Patients were restricted from visiting their healthcare providers due to supply shortages, staff on sick leave, and facilities that were at or above capacity and could not accept new patients due to a lack of resources [1,7,8,9,11,12,14,15,19,20]. Personal protective equipment (PPE) and supplies were in short supply and acted as a barrier to palliative care delivery because hospitals did not have enough supplies to accept an influx of COVID-19 patients [5,8,13,18] or had routine care suspended altogether for the unforeseen future. Hospitals, clinics, and home health teams even had to ration supplies to deliver palliative care services. Lastly, families and visitors were not able to provide support in-person during palliative care decisions due to these resource limitations and physical distancing requirements, also identified as a barrier to delivering high-quality palliative care [6].

### 4.5. Palliative Care Barrier: Accessibility of Care

Patients requiring sustained palliative care in the outpatient setting were greatly impacted during COVID-19 through a lack of accessibility to care, especially when local, regional, and even state-level shutdowns occur during the pandemic. Facilities closed and services were interrupted worldwide, with temporary and even long-term access to palliative care in the ambulatory care setting becoming nonexistent. In addition to decreased access to facilities, palliative care agencies faced challenges with in-home visits due to inadequate supplies, lack of personal protective equipment (PPE), and lack of staff [18,19]. The lack of emergent plans for how the outpatient care organization would function during a pandemic also became a barrier to access. Organizations had to disrupt the normal flow of clinic patient care and create emergency plans to continue to provide palliative care in some manner. The introduction of pandemic planning was designed to remove the barrier to access [1,17].

## 5. Conclusions

This comprehensive systematic review identified both facilitators and barriers related to the provision of palliative care in ambulatory care organizations located in the United States during the current COVID-19 pandemic. This study illustrates the unique challenges presented by the pandemic to patients requiring palliative care and to their providers. It also highlights unique steps and other potential processes/protocols to establish innovative manners of meeting the needs of this special population. It also identifies unique facilitators and barriers experienced by US outpatient organizations that may also be beneficial for other healthcare industries.

Many lessons learned highlighting best practices will come forth as a result of the pandemic. Specific to palliative care delivery, patient engagement, the benefit of advanced care planning, and the embracement of technology by both providers and patients are examples of successful constructs that are resulting in patient satisfaction and optimal health outcomes. Pandemic challenges related to palliative care identified in the research entail lack of resources such as telehealth equipment/training, insufficient bereavement/counseling resources for providers, and lack of access by certain patients. These challenges present areas for improvement in preparation for the next pandemic.

Future research surrounding this study’s findings include refining the capability to provide palliative care patients with more options to remain in their homes for this care and the identification of potential telehealth usage/implementation trends during the initial months of the pandemic. Future palliative care service lines should focus on the implementation of advanced care practices that promote physical distancing while enhancing the continuity of care to include an enhanced use of telehealth. While some research has been identified that identifies patient experiences related to palliative care adaptations during the pandemic, additional research surrounding patient clinical outcomes related to facilitated palliative care is an area for future research. Ambulatory care providers within and beyond the United States can benefit from identified palliative care sustainment facilitators and barriers as the global pandemic continues.

## Figures and Tables

**Table 1:** Reviewer assignment of the initial database search findings (full article review).

Article Assignment	Reviewer 1	Reviewer 2	Reviewer 3	Reviewer 4	Reviewer 5	Reviewer 6
Articles 1–7	X		X		X	X
Articles 8–14		X		X	X	X
Articles 15–21	X		X		X	X
Articles 22–28		X		X	X	X
Articles 29–32	X			X	X	X

**Table 2:** Summary of findings (*n* = 18).

Author(s)	Participant(s)	* JHNEBP StudyDesign	Facilitators of Palliative Care in Ambulatory Care Organizations during COVID-19	Barriers of Palliative Care in Ambulatory Care Organizations during COVID-19
Mishra et al. [1]	Outpatient cancer centers offering palliative care	4	Creation of a plan to control for resource limitation and personnel mobility restrictions Specialist palliative care centers need to coordinate and involve other primary-and secondary-level healthcare systems of their regions for providing a quality palliative care for all cancer patients when they are in need Admit (to inpatient) only those patients who require urgent in hospital palliative intervention Stockpiling adequate drugs, instruments, and resources Develop standard protocols for symptoms management Educate the patient and family Shift the care model to a family-centric one Build up communication skills and triaging priority patients for in-hospital and community-based palliative care services	Inadequate social support Lack of advanced care planning Inadequate information Fear of unknown disease Social distancing leads to difficulty in accessing family support for psychological, social, and physical symptoms management Poor peer support Limited visitor entry at home and hospices makes it difficult to maintain the integrity of family-centered care Difficulty in end-of-life care communication and shared decision making regarding advanced care planning due to physical distancing Difficult communication due to less face-to-face contact. Caregiver social distancing augments the feeling of loneliness, anxiety, and stress Limited access to telehealth due to lack of expertise in technology Economic stress due to loss of job Limited access to bereavement services Healthcare system (45) inadequate palliative care resources due to diversion for care of the large number of infected persons Quarantine of healthcare workers following an exposure also leads to shortage of workforce Ethical dilemmas in triaging resources Difficult access to opioids due to strict laws Psychological stresses, anxiety, fear of infection, insomnia, and posttraumatic stress disorder among healthcare workers Lack of personal support system for healthcare workers Difficulty in coordinating among multiple disciplines involved in cancer care Lack of adequate standardized protocol for different oncological treatment Lack of adequate hospices, especially in developing countries Lack of community-based healthcare workers during pandemic Unavailability of good network at remote areas for accessing teleconsultation
Jänig et al. [5]	US military medical treatment facilities	4	Palliative care was mentioned in the triage flowchart if a patient’s condition was worsening and there was not a realistic chance of improvement if they were transferred to an ICU; in this care, palliative care must be an option	N/A
Tran et al. [6]	Palo Alto Medical Foundation Palliative Care and Support Services	4	Creation of a triaging tool for patients needing palliative care during the pandemic maintained the safety and quality of care to other vulnerable populations Rapid transition of most consults to telehealth visits, triaging patients, and determining the risk associated by visiting/caring for patients in their home or facilities Frequent communication among interdisciplinary teams and use of the triage tool were found to be beneficial in keeping patients and staff safe while providing quality palliative care to those in need during the pandemic (patient scheduling benefit)	N/A
Roberts et al. [7]	Palliative care experts in primary care, including social work, pharmacy, nursing, and medicine at Johns Hopkins Bayview Medical Center (outpatient)	3	Weekly meetings included personal check-ins, creative brainstorming, candid peer-to-peer communication, and outcome-oriented organization and accountability Dissemination of a concise, user-friendly provider education that would enable primary care clinicians to identify potential management strategies for patients with COVID-19 who elect to remain at home for end-of-life comfort care Strong focus on organizational methods with creative nonhierarchical collaboration for outpatient providers Mnemonics and scripts as roadmaps identified as highly useful as providers care for their patients Study identified a lack of palliative care knowledge and resources available for outpatient primary care physicians, thus resulting in the development of a mobile app and toolkit to guide clinicians in discussions with COVID-19 patients electing to stay home for end-of-life services	Provider education gap identified in primary palliative care for outpatient clinicians during the COVID-19 pandemic Outpatient providers expressed communication gaps in protocols and best practices regarding palliative care
Cals et al. [8]	Primary care medical practices	3	The Consortium Research Family Medicine was started as a partnership among the 8 university departments to identify patients requiring palliative care during COVID-19 A registry database enabled providers to identify the number of positive COVID-19 patients or highly suspicious COVID-19 patients that died at home under the care of their general practitioner and whether they had received palliative care services from their primary care provider	Overcrowded ICU wards and hospitals were highlighted in the media, while outpatient providers were expected to provide continued palliative care to COVID-19 patients Access to care was determined to be inconsistent across the Netherlands, and, in some regions with the highest number of cases, there was more palliative care being done at home than in other areas
Lally et al. [9]	Department of Psychosocial Oncology and Palliative Care, Dana Farber Cancer Institute, Boston, MA	3	A palliative care clinic transitioned into a palliative telehealth clinic to continue/expand access to care The transition into palliative telehealth allowed for sicker patients to get easier access and stay protected during COVID-19 The telehealth transition during the pandemic enables a much higher level of interdisciplinary care among a wide range of medical providers Patients often initiated conversations about their goals and preferences at a higher level using telehealth resources to enable better palliative care during COVID-19 Increased scheduling flexibility was experienced by the clinic	Many regulatory, legal, and financial barriers to telehealth arose early in the transition Concerns such as lack of body language cues and the resultant difficulty in responding appropriately to emotion were difficult to assess via telehealth Some patients were unable to use video-based platforms, and phone became the only option for patients not wanting to risk an in-person encounter People without video access were forced to choose between the risks associated with an in-person visit versus a lower-quality telephonic palliative care visit
Trianti et al. [10]	Multiple outpatient clinic palliative care patients	2	The psychological impact of the pandemic in patients in the hospice appeared negligible. One could also argue that social isolation might conversely lead to a reduction in anxiety due to reduced input from the social environment.	However, patients under ambulatory palliative care reported measurable anxiety caused by COVID-19 that was comparable to the effect reported by the control population Considerable degree of anxiety experienced by the patients who visited the general practice of 4.5 out of 10 on the visual analog scale Patients under ambulatory palliative care experienced a similar (albeit moderate) degree of anxiety as patients visiting the general practitioner’s practice
Morris et al. [11]	Brigham and Women’s Hospital, Boston, MA, USA	4	Organizations can implement basic bereavement outreach, using palliative care tools and psychological strategies to prepare families for the death of their loved ones and to support them afterward in the initial months of their bereavement Care processes and communication skills identified as essentials services during palliative care in a pandemic	An urgency exists from a public health perspective to expand bereavement services in an attempt to mitigate poor bereavement outcomes
Oseni et al. [12]	Family practitioner clinics	4	Study identified that family practice physicians are often the first point of contact with COVID-19 patients; thus, incorporating the full spectrum of care to these patients at home can help mitigate the need to go to the hospital and help prevent the spread Efforts should be focused on educating and providing resources to primary care physicians in treating, mitigating, and offering end of life care to COVID-19 patients	Home visit and hospice care increase the cost of healthcare to patients and are mostly available to those who can afford it More flexibility of protocols suggested for disease management, training of caregivers, and providing appropriate technology including telemedicine to minimize the contact and promote social distancing
Pai et al. [13]	Staff nurses working in the wards/outpatient departments of a reputed palliative care center	4	Nurses providing palliative care experienced the following facilitators: Counseling and emotional support Appreciation and reward Use of telenursing services	Nurses providing palliative care experienced the following barriers: Fear of acquiring infection and risking own health Fear of harming family members or losing loved ones Changes in sleep, eating patterns, and concentration issues Worsening of chronic health problems Fear of avoidance from the community Access to palliative care services
Arya et al. [14]	Multi-specialty clinics	4	Providers advised to stockpile medications and supplies used in palliative care, train staff to meet palliative care needs, optimize space, refine systems, alleviate the effects of separation, have critical conversations, and focus on marginalized populations to ensure that all patients are cared for equitably Preparing and distributing sufficient numbers of “palliative medication kits” could help address this issue in any setting with substantial numbers of patients who might not survive. Have spiritual care staff and social work ready to manage common psychosocial needs such as grief and bereavement	Palliative care services are needed across many different care settings, including intensive care units, hospital wards, emergency departments, and long-term care centers (access issues) In a pandemic, patient autonomy to choose life-prolonging measures or location of death could be severely restricted as a result of public health directives and resource availability Some patients are isolated at end-of-life stages
Inzitari et al. [15]	Post-acute facility	3	Advanced care planning with reasonable therapeutic effort for each patient facilitated palliative care Accelerated screening of patients and staff through polymerase chain reaction (PCR) tests Acute care treatment of COVID-19 was balanced with palliative and geriatric care, mainly oriented toward preventing and managing delirium using nonpharmacological interventions	Instituted lockdown to external visitors
McKenna et al. [16]	Advanced care planning initiative	3	Three advanced care planning key themes were identified: (a) feeling emotionally safe enough to have such sensitive conversations is vital; (b) participating in the HLD process increases the confidence of those participants who worked in health and social care, to undertake ACP conversations; (c) planning ahead is a complex, staged process rather than a single record-making event This article identified advanced care planning as a facilitator to effective care during the pandemic	N/A
Wei et al. [17]	New York City Health + Hospital system pandemic established protocols	4	Provision of daily updates by telephone and used tablets for virtual visits Expanded palliative care team held virtual consultations with families to discuss advance care planning and end-of-life decisions	N/A
Seminara et al. [18]	Medical home visit program embedded in the Divisionof Geriatrics at Staten Island University Hospital in New York	4	Benefits of telehealth for families and patients were identified in the article Defining the most critical cases first proved beneficial Establishment of pre-visit and post-visit protocols for palliative care Addressing staff concerns surrounding motivation and honesty of issues experienced, and identifying most vulnerable patients during the pandemic	Fear of encountering COVID-19 precipitates poor decision making Inadequate supplies of timely diagnostics for COVID-19 adversely affect services for homebound patients A lack of personal protective equipment also diminished services by vendors such as visiting nurses and physical therapists COVID-19 triggered altered end-of-life decisions by those afraid to be without loved ones in the hospital
Page et al. [19]	Home-based care strategy and experience of the Cipla Palliative Care and Training Center	4	N/A	Difficulty in accessing medical care in the event of increased symptom burden Obstacles in reaching hospitals at time of emergencies or end of life Limited access to medication, and social distancing causing isolation, leading to psychosocial burden Lack of bereavement support
Cheng [20]	Medicine and geriatrics department	4	Caregivers respond rapidly and flexibly Caregivers ensure protocols for symptom management are available, considering redeploying staffs and volunteers to provide psychosocial and bereavement care and using technology to communicate with patients and providers Mobile consultative team should be available in situation of difficult symptoms control, for instance, in cancer patients who were already receiving opioids for pain, or when goals of care became unclear in advanced cancer patients	Immunosuppressed status of some cancer patients, whether caused by the disease itself or the treatment, increases their risk of infection compared with the general population
Weinstein et al. [21]	Penn Medicine Head and Neck Cancer Service Line	4	Ethical care guidelines guided by professionals and experts in this field, including inpatient and outpatient care for patients with head and neck cancers Extension of medication treatment to prevent frequent in-person visits Consideration for decreased frequency of in-person visits and imaging studies Telehealth should be utilized for end-of-life care and palliative care through Penn health Much or all of end-of-life care can be delivered at home via telehealth visits	N/A

* Johns Hopkins Nursing Evidence-Based Practice (JHNEBP) levels of strength of evidence: level 1, experimental study/randomized control trial (RCT); level 2, quasi-experimental study; level 3, nonexperimental, qualitative, or meta-synthesis study; level 4, opinion of nationally recognized experts based on research evidence/consensus panels; level 5, opinions of industry experts not based on research evidence.

**Table 3:** Summary of quality assessments.

Scheme 1	Frequency
2 (Quasi-experimental)	1 (5.5%)
3 (Nonexperimental, qualitative)	5 (28%)
4 (Opinion of nationally recognized experts based on research evidence/consensus panels)	12 (66.5%)
5 (Opinions of industry experts not based on research evidence)	0 (0%)
